# Deep-sea benthic communities in the largest oceanic desert are structured by the presence of polymetallic crust

**DOI:** 10.1038/s41598-019-43325-0

**Published:** 2019-05-06

**Authors:** Juliette Delavenne, Louise Keszler, Magalie Castelin, Pierre Lozouet, Philippe Maestrati, Sarah Samadi

**Affiliations:** 1 Institut Systématique Evolution Biodiversité (ISYEB), Muséum national d’Histoire naturelle, CNRS, Sorbonne Université, EPHE, CP 26, 57 rue Cuvier, 75005 Paris, France; 2 Unité Biologie des Organismes et Écosystèmes Aquatiques (BOREA UMR 7208), Sorbonne Université, MNHN, Université de Caen Normandie, Université des Antilles, CNRS, IRD, CP26, 57 Rue Cuvier, 75005 Paris, France; 3 Centre d’Ecologie et des Sciences de la Conservation (CESCO UMR7204), Sorbonne Universités, MNHN, CNRS, UPMC, CP51, 57 rue Cuvier, 75005 Paris, France; 4 Direction des Collections, Muséum national d’Histoire Naturelle, CP51, 57 rue Cuvier, 75005, Paris, France

**Keywords:** Biodiversity, Biooceanography

## Abstract

Based on the specimens collected during three deep-sea cruises, and deposited at the Muséum National d’Histoire Naturelle (MNHN) in Paris, we analysed the diversity of benthic communities within the EEZ of French Polynesia. The literature and the MNHN database allowed us to inventory 471 species of invertebrates, among which 169 were newly described. We mainly found data for Mollusca, Crustacea, Brachiopoda and Crinoidea. We also found samples from other taxa, which still remain unidentified within the collections of the MNHN. Although this inventory is incomplete, we demonstrate that the deep waters of French Polynesia host unique benthic communities and endemic species. Using diversity and multivariate analyses, we show that the deep-sea benthic communities are structured by depth, habitats, geography and also by the presence of polymetallic crust. Furthermore, by focusing on the molluscs of the central area of French Polynesia, we show that the spectrum of shell size differs among deep-sea habitats. Specifically, shells tend to be smaller on encrusted seamounts than on island slopes. Together with the size range of organisms, low abundance, rarity and endemism designate these habitats as sensitive. These results should thus be taken into account in the evaluation of the expected impact of mining activities on biological communities.

## Introduction

Deep-sea waters represent more than 90% of the habitable volume on earth (Levin and Le Bris 2015), and host the most poorly known habitats on earth^1^. In the context of such poor state of knowledge, the exploration of the deep sea for new mineral resources calls for more data on its biological diversity^2^. Mining activities critically modify the benthic habitats, both by removing substrata on which organisms are living, and by modifying environmental factors. Specifically, an increase of sedimentation rate resulting from the release of mining residues is expected^3,4^. In French Polynesia’s Exclusive Economic Zone (EEZ), a recent interest for the mining potential of thick polymetallic crusts, fortuitously discovered on some of its seamounts, raised the question of the vulnerability and resilience of associated biological communities^5^. Contrary to massive sulphide deposits associated with active or inactive hot-vents^6^, or to polymetallic nodules in very deep abyssal plains^7^, the benthic communities on seamounts with polymetallic crust are still poorly studied. The available data mainly concern the seamounts in the Central North Pacific^8,9^. In the deep sea, seamounts, generally defined as topographic features raising from at least 1,000 meters from the seafloor, represent ubiquitous features, but only a very few of them have been studied^10^. Most of these features, including those of the French Polynesian EEZ^11^, have a volcanic origin. Seamounts disrupt the movements of the oceanic water masses. This results in lower sedimentation rates, and an enhanced delivery of food particles to the suspension-feeding animals, in comparison to other deep-sea habitats such as abyssal plains or continental margins^12^. The biological communities on hard-substrata consist of suspension-feeding organisms (such as hard corals, gorgonians, sponges or pedunculated crinoids) which typically host a diverse set of associated fauna^13^. Polymetallic crusts also build up on such hard substrates, depleted of sediments for millions of years^14^.

**Figure 1:**
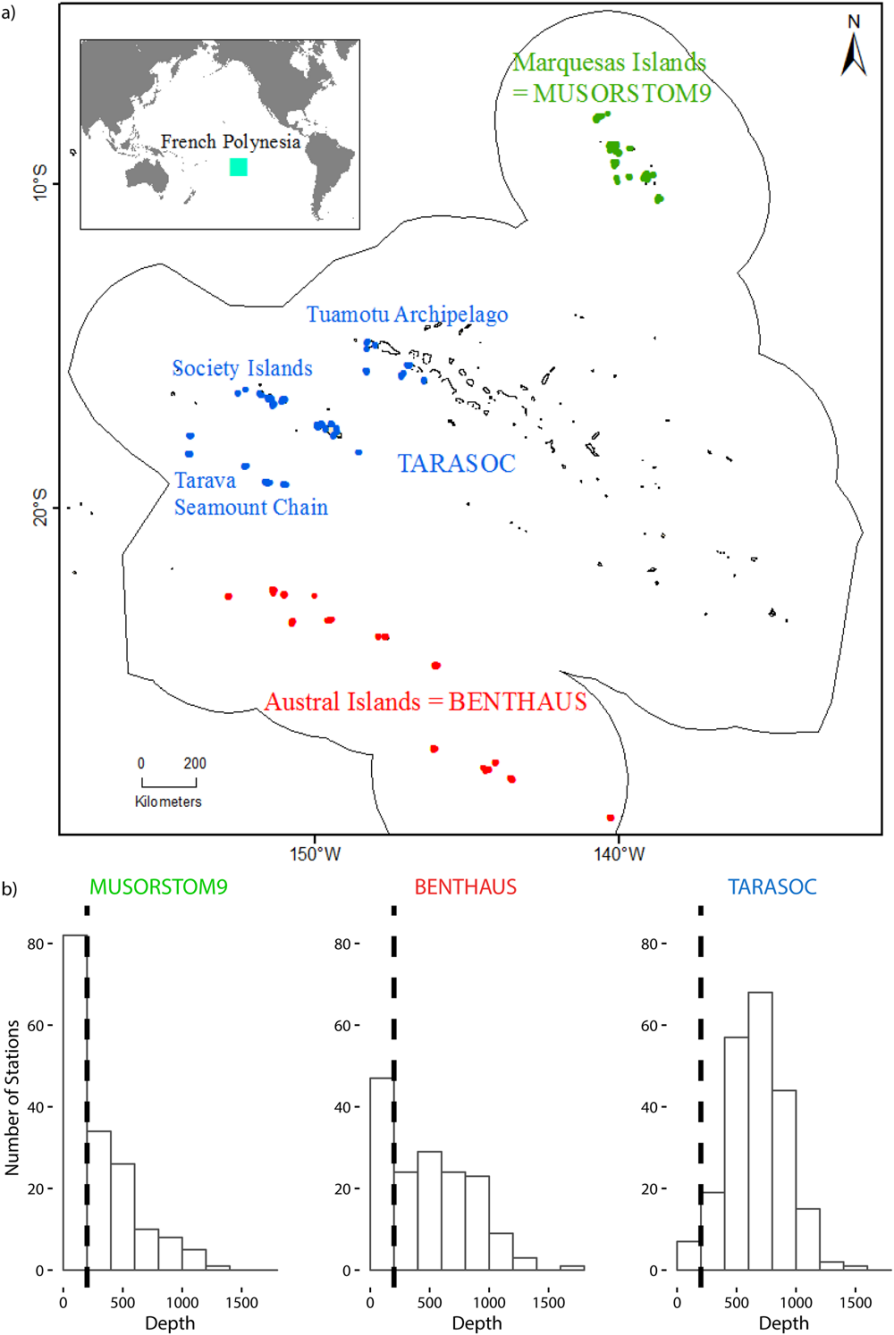
(**a**) French Polynesia. The sampling sites of the three TDSB surveys: MUSORSTOM 9 (green), BENTHAUS (red), and TARASOC (blue). (**b**) Depth distribution of the sampling sites for the 3 surveys, the black dashed line is the 200 m depth limit.

So far, the biological communities associated with the hard bottoms of the central Pacific region (specifically the EEZ of French Polynesia) have remained unstudied. French Polynesia spreads over 20° longitude and covers 4.7 million km² (Fig. 1a). The various archipelagos found in this EEZ have distinct geological histories, and unique oceanographic and climate regimes^15,16^. Among the specific features of the area, it is worth noting that French Polynesia is isolated in the central Pacific, in the middle of the South Pacific Gyre, often considered as the Earth’s largest oceanic desert from the sea surface to the deep sea floor^17^. Within this context, our aims were (i) to gather available data on the deep-sea benthic communities of the French Polynesian EEZ; and (ii) determine if the observed polymetallic crusts, occurring mostly on seamounts, are associated with unique benthic communities.

The sole cohesive dataset available for the analysis of the biodiversity of the deep-sea benthic communities in the area is provided by the sampling of the Tropical Deep-Sea Benthos (TDSB) program^18^. TDSB is an informal program driven by a naturalist exploration approach aiming at discovering new taxa, and at characterizing their range of geographic distributions. The majority of the data provided by these expeditions are in the form of biological specimens, which are deposited at the Muséum National d’Histoire Naturelle (MNHN) in Paris. Taxonomic identifications for these specimens can be found primarily in the published taxonomic literature, while unpublished identifications (or records) are available from the MNHN databases. Three TDSB cruises were carried out in French Polynesia (Fig. 1a): MUSORSTOM 9 (1998), BENTHAUS (2002) and TARASOC (2009). Parallel to these three TDSB surveys, a consortium of French and Polynesian institutes launched a suite of oceanographic campaigns under the umbrella of the Zone Economique de la POLYnesie Française (ZEPOLYF) program^19^. The aims of these campaigns were to chart the seabed of the French Polynesian EEZ, and to make an inventory of the mineral and biological resources within the area. For this study, we used the largest biological dataset available (comprising data for crustacean, molluscs, brachiopoda and crinoidea) and all available geological data for the EEZ. Our first purpose was to determine whether geography, depth, topography and substrate types, structure the benthic communities at the scale of the EEZ. Then, to evaluate the characteristics of the communities established on polymetallic crust, we focused on the molluscs collected during the TARASOC survey. This cruise explored an area potentially targeted by the exploration for mineral resources. The molluscs were studied because the number of specimens per sampling unit is greater than for other taxa. Moreover, their shells provide an easy way of comparing the different communities, not only in terms of diversity, but also in terms of functional traits. In benthic communities of the deep sea, the quality and quantity of food may affect the presence, and relative abundances, of species from different size-classes, with consequences on the trophic structures within communities^20,21^. In this context, the shell size allowed us to infer the body size and thus to compare the communities for this functional trait. We finally discuss our results in the context of the economic interest for deep-sea mineral resources in this region.

## Methods

### Study area and deep-sea surveys

In 1998, the MUSORSTOM 9 cruise explored bathyal slopes of Marquesas. Most of the islands had abrupt steep slopes starting around 100 m water depth, with some terraces between 400–600 m depth. As the R/V ALIS, used for most TDSB cruises, was not yet equipped with a multibeam echo-sounder, uncharted zones were poorly explored and a single seamount was visited (see Fig. 1a). In 2002, the BENTHAUS cruise explored slopes and seamounts of the Austral Islands. In 2009, the TARASOC cruise explored both the bathyal slopes of the Society and Tuamotu Islands, and the Tarava Seamounts chain. For these two cruises, data from the ZEPOLYPH cartography program (1996–1999), and a multibeam echo-sounder, newly available at that time on board the R/V ALIS, allowed sampling on deeper, steeper, and more rugged terrain, which are typically found on seamounts (Fig. 1b). As the three TDSB cruises correspond to three distinct geographical areas, the names of the cruises are used throughout the text to compare the three areas to one another (Fig. 1a).

The sampling plan of this naturalistic program aims at maximising the diversity identified. Because each type of gear does not catch the same fauna, the deployment of every gear type is attempted when possible at each locality. However, at localities dominated by hard and uneven bottoms, it is not always possible to deploy trawls. Overall, the three cruises represented 539 sampling events (stations), mainly with dredges or trawls, of which 391 were carried out below 200 m (Table 1). The deepest station was 1,800 m depth (Fig. 1b). The sampling covered the slopes of 25 islands and the summits of 13 seamounts (Fig. 1a). TDSB sampling metadata are available on the MNHN expedition website: https://expeditions.mnhn.fr. This database provides metadata for each station, such as the geographic coordinates and the sampling depth and if available, photos taken during the sorting process on board. All the sampled organisms are labelled on board with a station number and are available for research to an international network of taxonomists. After taxonomic identification, specimens are registered into the MNHN database, and linked to station data provided by the expedition database. Note, however, that some of the identifications that have been published in scientific papers, are not yet registered in MNHN database and, conversely, some of the identifications that are registered, have not yet been published in scientific papers.

**Table 1:** Sampling events (stations), sampling gears, number of families and species sampled during the TDSB cruises.

		MUSORSTOM9	BENTHAUS	TARASOC
Sampling events (stations)	**Total**	**166**	**160**	**213**
	Below 200 m	82	107	202
Gears	Beam Trawl (CP)	31	12	24
	Waren Dredge (DW)	27	93	178
	Rock Dredge (DR)	24		
	Traps (CAS)		2	
Number of families	**Total**	**70**	**61**	**66**
	Mollusca	30	27	35
	Crustacea	38	30	21
	Crinoidea		1	4
	Brachiopoda	1	3	5
Number of species	**Total**	**137**	**202**	**229**
	Mollusca	69	92	125
	Crustacea	67	102	89
	Crinoidea		4	10
	Brachiopoda	1	4	5

### Species occurrences data

We compiled a list of occurrences of benthic invertebrate species below 200 m depth in the French Polynesian EEZ (see Supplementary Material) using a variety of sources. First, we reviewed all the literature concerning samples from the MUSORSTOM9, BENTHAUS and TARASOC cruises (the list of publications used for each cruise is available at expeditions.mnhn.fr). Second, we cross-checked the species occurrences against the information available within the MNHN collection database (https://science.mnhn.fr), and the Joseph Poupin’s crustacean database (http://crustiesfroverseas.free.fr/). This enabled us to add: (1) species that are currently under description (referred to as sp. *nov*.); (2) known species that had been identified by taxonomists as occurring in French Polynesian EEZ, but for which the records have yet to be published.

**Figure 2:**
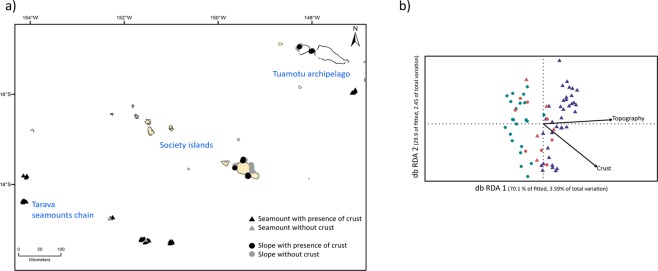
(**a**) The selection of the 74 sample sites from the TARASOC survey for which the molluscs, extracted for the small sieve residuals, were sorted into morphospecies. This set of stations was used to generate the dataset #2. (**b**) Distance-based RDA ordination plot visualizing the relationships between the benthos communities from dataset #2 and two environmental constraints: topography (Seamount or Slope, p-value = 0.001) and crust presence (p-value = 0.6). Seamounts are represented by triangles and slopes by circles. The colours of the symbols indicate the region sampled during the TARASOC cruise: green represents the Society Islands, red represents the Tuamotu Archipelago, and blue represents the TARAVA Seamounts.

After reviewing the literature and databases, we compiled the data over the three surveys, for the Mollusca, the Crustacea, and the Brachiopoda. For other taxa, such as Cnidaria, Echinodermata, Porifera and Vertebrata (i.e. fishes), we found occurrences to be fewer than ten species, or we only found species occurrences for one of the three surveys. Third, we explored the MNHN collections for unidentified and/or unregistered specimens. We found important amounts of unstudied material, mostly for Cnidaria, Porifera, Echinodermata, Vertebrata (fishes) and Mollusca; but with the exception of Crinoidea and Mollusca, no experts were available to identify these specimens. Nadia Améziane and Marc Eleaume, kindly accepted to sort the crinoids from the three surveys into morphospecies. For Mollusca, contrary to the other cruises, the residues from TARASOC were fixed in ethanol on board, and were then sorted by Michel Boutet, who identified the specimens at the family level^22^. Mollusca extracted from residues are mostly smaller than 5 mm and, following Albano *et al*. (2011), are named micromolluscs^23^. These micromolluscs represent a rich source of taxonomic information. Given the huge amount of material, we selected a subset of stations covering the Tarava seamounts, the Tuamotu seamounts and slopes, as well as the slopes of the Society Islands (Fig. 2a). Unidentified specimens sampled at these stations were sorted into morphospecies (*i.e*. one or several specimens are hypothesized as being a distinct species, on morphological characteristics only, without formally being described or given a name^18^). The micromollusc Turridae were not included in the morphospecies sorting as it represented too much material. Moreover, the assessment of the species diversity based on morphological traits for this group is difficult to establish^24^.

Last, we made an additional quality control for shelled molluscan species. Empty shells may be carried from shallow to deeper seafloor, and may not be representative of the depth at which the animals are living. Therefore, molluscan species recorded from the literature or in our specimens, occurring at shallow depths (0–200 m), but also present as dead shells below 200 m, were considered as belonging to shallow-waters species and thus discarded from the final dataset.

### Environmental data

The presence of polymetallic crusts was established for each sampled locality (geomorphological features such as islands or individual seamounts) based on the reports from the geological surveys in the area. Four surveys provided information about the presence of polymetallic crusts. The NODCO (1986) cruise discovered the first site with thick crusts on a seamount in the Tuamotu Archipelago. The Tarava Seamount Chain was discovered during ZEPOLYF1 (1996) cruise. The POLYDRAG (1998) cruise then sampled the crusts and complemented the bathymetric maps. Last, the ZEPOLYF2 (1999) cruise surveyed the Austral Islands^11^. Since the sampling area of BENTHAUS and ZEPOLYF2 overlapped, the presence of polymetallic crusts for BENTHAUS stations were inferred from the ZEPOLYF2 results^16,25^. For the TARASOC cruise, we complemented the available geological data using the photos of the content of the trawls and the dredges. These photos, taken on board of the R/V ALIS, allowed us to assess, for each station if crusts were present. The cruise report of MUSORSTOM 9 was used to determine if the sampled sites in Marquesas were encrusted^26^.

### Data analyses

In order to characterise the deep-sea benthic communities of French Polynesia, we defined two datasets corresponding to two distinct geographic scales and taxonomic coverage. Dataset #1 encompasses all occurrence data over the area covered by the three cruises, (not including micromolluscs from the TARASOC cruise). We grouped the stations by localities corresponding to geomorphological features, such as islands or individual seamounts, and defined regions corresponding to the three surveys (see Fig. 1a). For dataset #2, we focused only on molluscs from the set of stations of the TARASOC cruise (Fig. 2a). For each species of the dataset #2 we recorded the size, by either measuring the biggest specimen, using a digital calliper, or we used the size documented for the holotype. The availability of shells within the sieve residues also allowed us to compare, for this subset of stations, the diversity of the communities of micro- and macro-molluscs.

All analyses were carried out with a species by localities (dataset #1) matrix or a species by sampling sites (dataset #2) matrix, in R using the packages *vegan*^27^, *iNEXT*^28^ and *recluster*^29^. To compare diversities among localities that were not evenly sampled, we evaluated species and families richness using sample-based rarefaction and extrapolation curves with Hill Numbers^30,31^. Species richness was compared among the three surveys (MUSORSTOM 9, BENTHAUS, and TARASOC) for dataset #1, and between habitats (Seamount *vs* Slope) for the dataset #2. For dataset #1, we used a Venn diagram to visualize the amount of species shared among regions. For dataset #2 we analysed separately the richness of the micro- and the macro-molluscs.

For both datasets, we computed a similarity matrix using the Sørensen - coefficient. For dataset #1, first, non-metric multidimensional scaling (nMDS)^32^ allowed us to visualize similarities of communities among the different localities. Then, for both datasets, we tested the influence of environmental variables (e.g., the mean depth and the presence of crusts for dataset#1, and the topography and the presence of crust for dataset #2) on the structure of the communities using a distance-based Redundancy Analysis (db-RDA)^33^. Finally, we conducted PERMANOVA^34^ –analyses. This test is used to test the significance of the variance of the dissimilarity in the composition of communities. We looked at the dissimilarity, among the regions and habitats for dataset #1, and among the regions, the habitats, and the life history traits for dataset #2.

## Results

**Figure 3:**
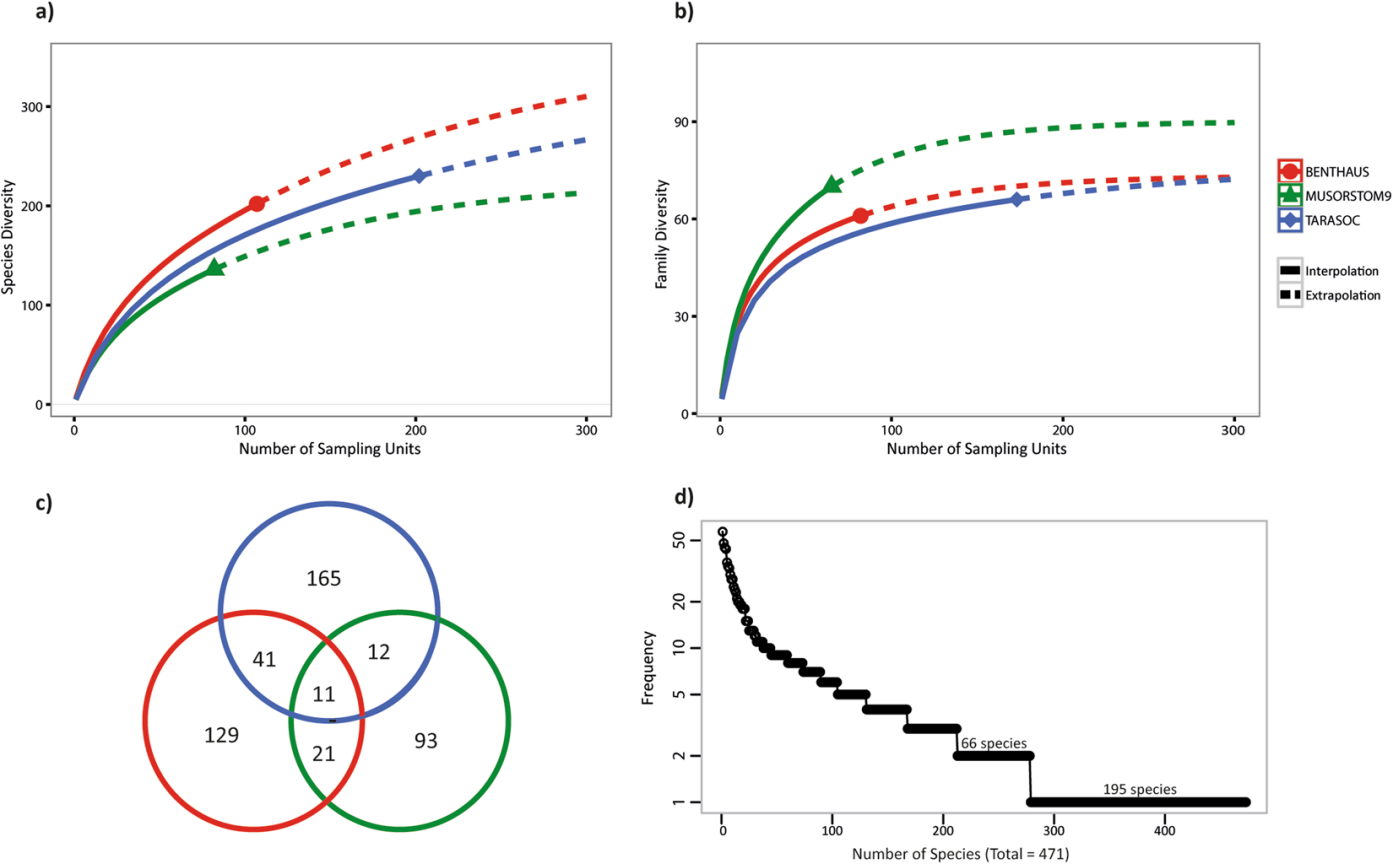
(**a**) Rarefaction (solid line) and extrapolation (dashed line) curves illustrating estimate of the species richness in the dataset #1. (**b**) Rarefaction (solid line) and extrapolation (dashed line) curves illustrating the families’ richness in the dataset #1. (**c**) Venn Diagram presenting the number of species shared by the three surveys. (**d**) Rank frequency diagram of the species present in the dataset #1.

Over the three cruises, 471 species belonging to Mollusca, Crustacea, Echinodermata and Brachiopoda were included in dataset #1 (Table 1). More dredges were deployed for the BENTHAUS and TARASOC cruises, during which, more seamounts were explored than for the MUSORSTOM9 cruise. However, among the three cruises, the relative numbers of mollusc species (better sampled with dredges) and crustacean species (better sampled by trawls or traps), were similar. BENTHAUS was the most species diverse of the three surveys (Table 1 and Fig. 3a). By rarefying the number of sampling events to a comparable number (80), we obtained an estimated species richness of 175 for BENTHAUS, 135 for MUSORSTOM 9, and 153 for TARASOC (Fig. 3a). Focusing on a higher taxonomic rank (e.g. Family), MUSORSTOM 9 was the most family-rich survey (Table 1, Fig. 3b). The Venn diagram (Fig. 3c) shows that 2.3% of the whole diversity was shared among the three surveys, and that more species were shared between MUSORSTOM 9 and BENTHAUS (21) than between MUSORSTOM 9 and TARASOC (12).

**Figure 4:**
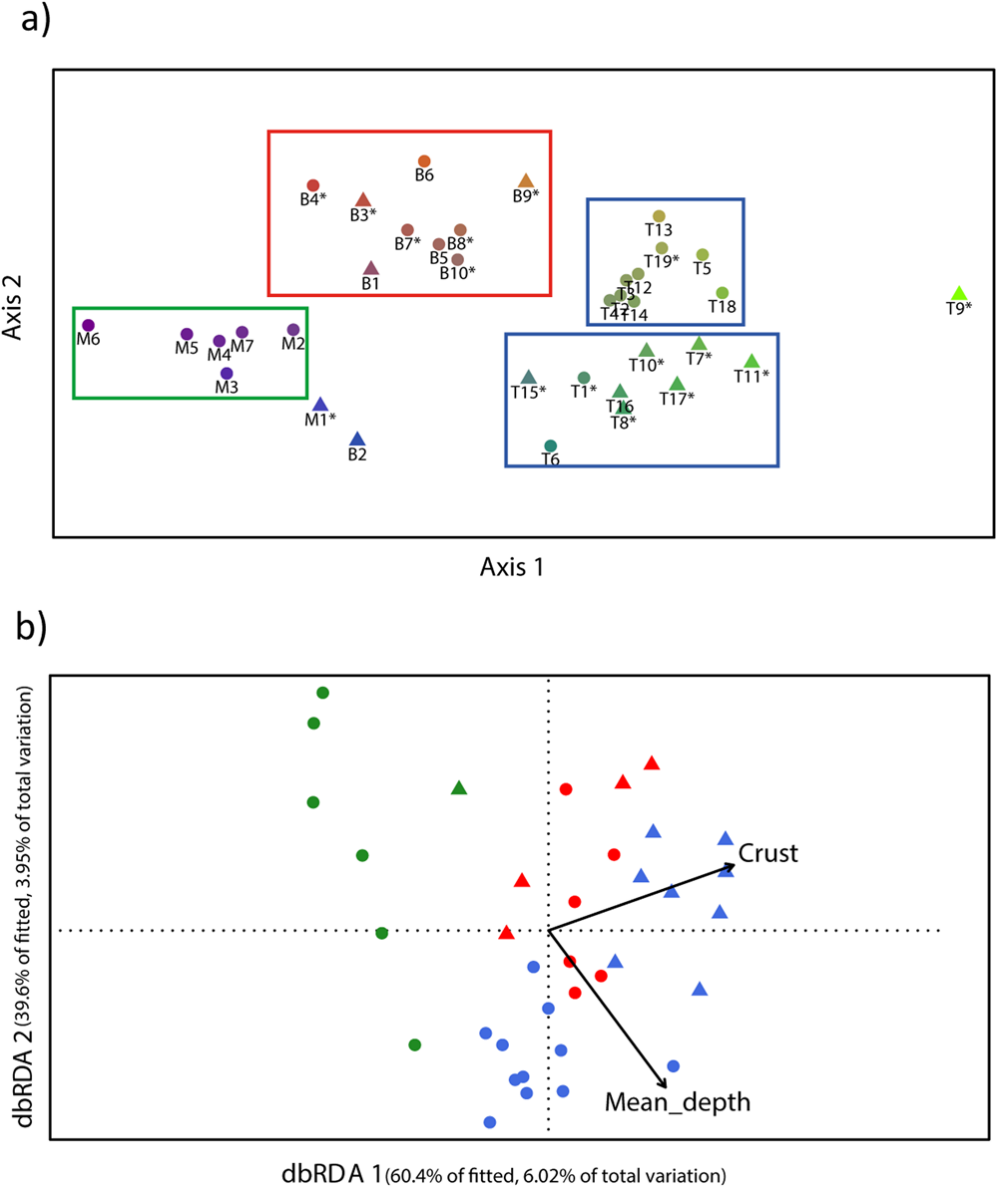
(**a**) nMDS ordination plot for dataset #1, showing the benthic community similarities among samples from the localities of the three surveys (stress = 0.18). The colours of the symbols represent the similarity between the communities; B stands for BENTHAUS, M for MUSORSTOM 9 and T for TARASOC. The localities with polymetallic crusts are identified with a star. Seamounts are represented by triangles and slopes by circles. BENTHAUS localities are grouped in a red polygon, MUSORSTOM 9 localities are grouped in a green polygon. TARASOC localities are grouped in two blue polygons: one for the seamount localities and one for the slope localities. (**b**) Distance-based RDA ordination plot visualizing the relationships between the benthos communities from dataset #1 and two environmental constraints: depth and crust presence (p-value = 0.015 and 0.009, respectively). Seamounts are represented by triangles, and slopes by circles. The colours of the symbols indicate the cruise: red for BENTHAUS, green for MUSORSTOM 9 and blue for TARASOC.

Three groups were distinguished in the nMDS plot obtained for dataset#1 (Fig. 4a): (1) localities from the MUSORSTOM 9 survey (green), (2) localities from the BENTHAUS survey (red), and (3) localities from the TARASOC survey (blue). The TARASOC group presented two sub-groups corresponding to islands slopes (circles in the top box), and seamounts (triangles in the bottom box). Three points fell outside of these groups, corresponding to localities with very low species richness; even though the number of stations per locality was of the same order than for the other localities. The PERMANOVA test showed that species assemblages were significantly different among the three cruises (F statistic = 0.001). Both the mean depth of the sampled locality, and the presence of crust, significantly structured the species assemblages (p-value = 0.015 and 0.009, respectively; db-RDA plots, Fig. 4b).

The dataset #2 included 287 molluscan species (of which 216 are morphospecies from the TARASOC residues), over 74 sampling sites of the TARASOC cruise: 155 species of micromolluscs and 132 species of macromolluscs. Molluscan communities were structured by both topography (Seamount or Slope, p-value = 0.001) and the presence of crust (p-value = 0.001) (db-RDA, Fig. 2b).

**Figure 5:**
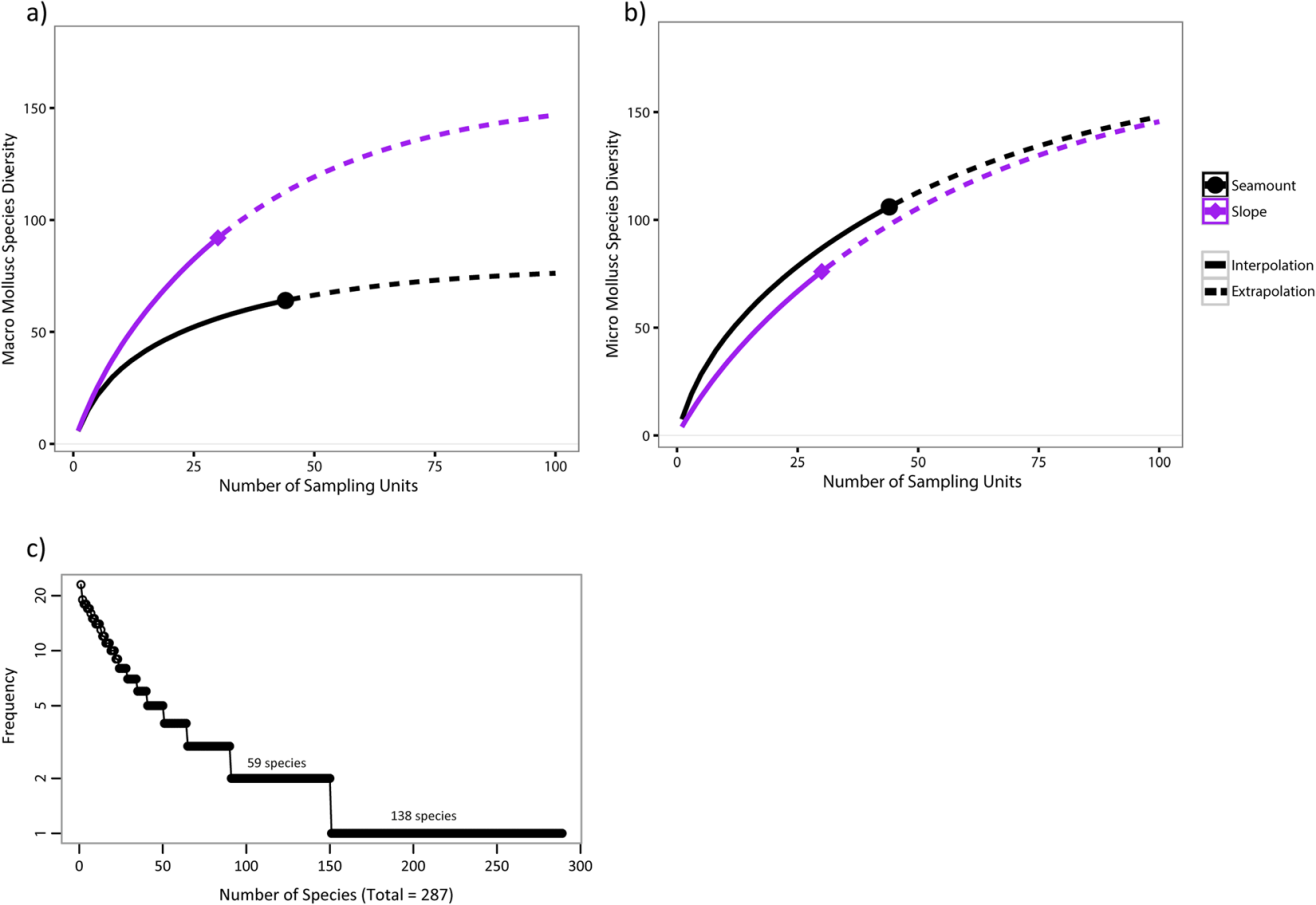
(**a**) Rarefaction (solid line) and extrapolation (dashed line) species accumulation curves for dataset #2. Estimated species richness are compared between the seamounts and slopes sampling sites for (**a**) the macromolluscs (>5 mm) and (**b**) the micromolluscs (<5 mm). (**c**) Rank frequency diagram of the micro- and macromollusc species included in the dataset #2 (287 species in total, n = 155 for the micromolluscs and n = 132 for the macromolluscs).

For macromolluscs, the slopes were more species-rich than seamounts. For example, for 24 sampling events, the curves indicate an estimated species richness of 80 and 51, respectively, for slopes and seamounts, whereas for micromolluscs, no difference in species richness between these two environments, could be observed (Fig. 5a,b). Moreover, species assemblages on slopes and seamounts were significantly different (PERMANOVA Fstat = 0.001) for both macro- and micro-molluscs. Likewise, significant differences in assemblage composition were associated with the presence of polymetallic crust for both size groups (PERMANOVA Fstat = 0.001 and 0.008 respectively).

**Figure 6:**
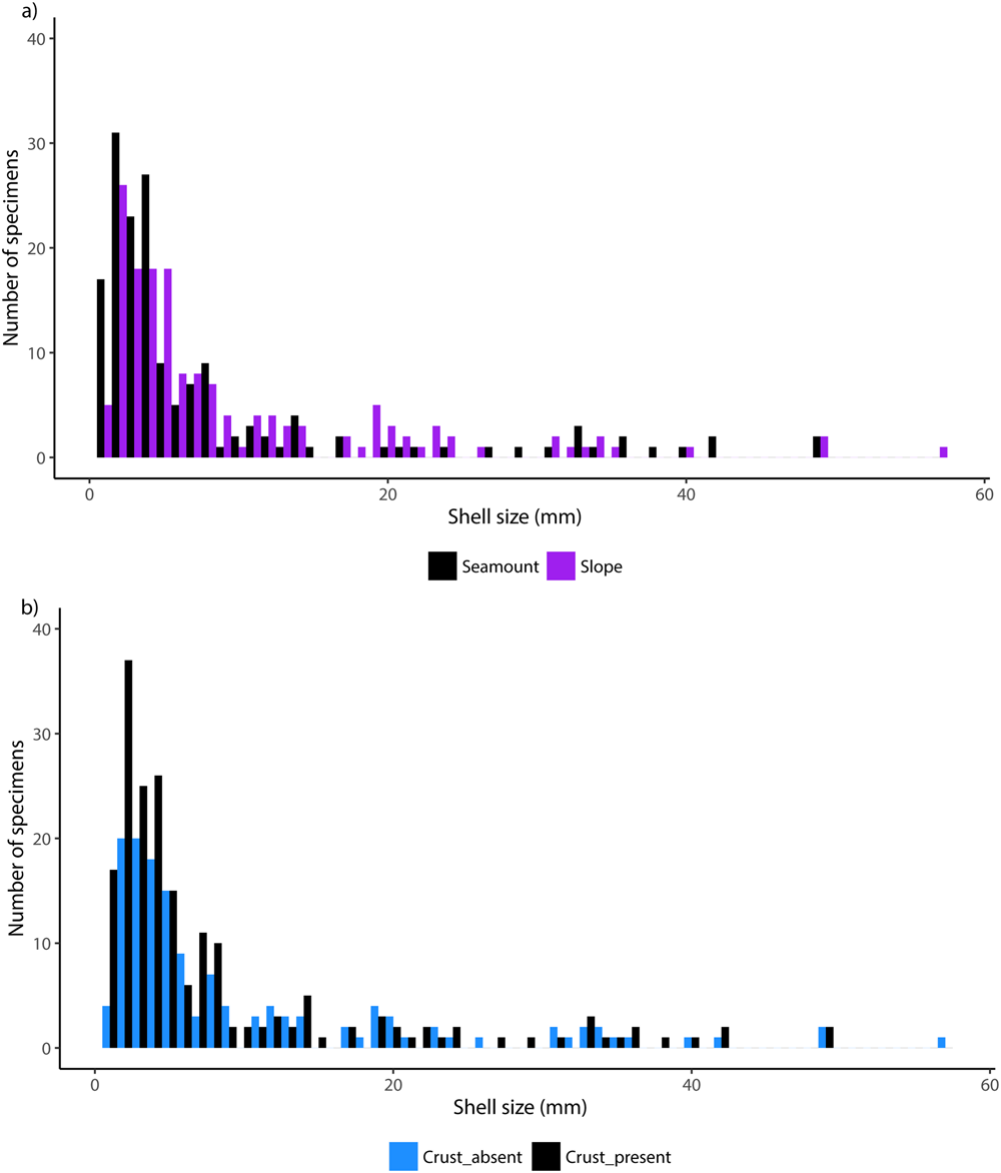
(**a**) Distributions of the shell sizes (mm) of shelled molluscs from dataset #2 for sample sites on seamounts and slopes (Kruskall Wallis, pvalue = 0.012). (**b**) Distributions of the shell sizes (mm) of shelled molluscs from dataset #2 for sample sites with crust and without crust (Kruskall Wallis, p-value = 0.021).

Finally, the distribution of shell sizes was significantly different between seamounts and slopes, (p-value = 0.007, Kruskal-Wallis test), with more micromolluscs found on seamounts (Fig. 6a). Moreover, the distribution of the size of the shells according to the presence of polymetallic crust showed that more shells were smaller than 5 mm on crust (p-value = 0.021, Kruskal-Wallis test, Fig. 6b).

## Discussion

The naturalist surveys carried under the umbrella of the Tropical Deep Sea Benthos program (TDSB) provide the only cohesive data set to describe the structure of deep-sea biodiversity in French Polynesian EEZ. Based on the results of the three TDSB expeditions, we inventoried 471 invertebrate species. Among these 471 species, 169 are newly described species based on TDSB sampling, and 30 are either under description, or not yet attributed to a species name. This high proportion of species newly described, together with the amount of material still left to be examined by taxonomists, stresses how much diversity remains to be explored for these environments, especially in such remote areas. Nonetheless, the analyses of these data allowed us to provide a first insight into the biodiversity patterns of the deep-sea benthic communities of French Polynesia, in a context of potential deep sea mining activities.

French Polynesia is isolated in the middle of the South Pacific Gyre, at the tail of a species richness gradient, in which the core is represented by the Indo-Australian Archipelago (Coral Triangle^35–37^). The strong turnover in species composition, from the Coral Triangle to the Central Pacific, reflects the higher level of endemic species often observed in the region^36,38^. The marine diversity of French Polynesia is lower than in the western regions of the South and Central Pacific^35,39,40^. The few studies that examined species turnover on large scales for deep-sea taxa also observed this trend. For example, Macpherson *et al*. (2010) identified in the area 46 deep-sea squat lobster species, of which 78% were endemic to the French Polynesian waters^41^. With the additional data gathered from this study (59 instead of the 46 species in Macpherson *et al*. 2010), this value is revised to 70%, which is still a high proportion of endemics. Furthermore, these endemic galatheid species are equally distributed over the three prospected areas, and over the different explored habitats. Cone snails represent another example. In our dataset, among the 22 species of Conidea sampled below 200 m in French Polynesia, 30% can be considered as endemic to this area. However, this number should be taken with caution since recent studies showed that, among the listed species, some belong to species complex pending revision. For example, based on available literature data, we considered *Conus orbignyi* as cosmopolite. However ongoing molecular studies revealed that under the name *C. orbignyi*, a complex of at least nine species is concealed, some of them being geographically limited to one archipelago^42^. Since, only dead shells of cone species were collected in the deep waters of French Polynesia, it is likely that our study underestimates their endemicity.

Results tend to show that the deep-sea communities of French Polynesia have an especially high score of endemism and rarity. Rarity is a relative concept. Usually, the species falling in the first quartile of the frequency distribution of species occurrences are considered as “rare”^43^. Here, 40% of the species in the dataset #1 were only sampled once (Fig. 3d). Hence, it would be meaningless to apply the 25% cut-off to this dataset. Moreover, the species that were sampled only once were often the ones sampled with only a few specimens. The interpretation of the data, in terms of endemism, may thus be obstructed by the apparent rarity of most sampled species, resulting from the shortfall of sampling over such a large area. Indeed, our sampling is not saturated for any of the three regions (Fig. 3a), and the heterogeneity of the upper bathyal region of the whole French Polynesia is far from covered with 391 stations.

In addition to the limited number of sampling stations in relation to the size of the French Polynesian EEZ, it is also worth considering the taxonomic coverage of the available dataset. Indeed, although all the phyla have been collected during these cruises, only the Molluscs, Crustaceans, Crinoids and Brachiopods have been extensively identified. It should be noted that Bertrand Richer de Forges, the PI of MUSORSTOM 9 and BENTHAUS cruises is a carcinologist and that Philippe Bouchet, the PI of TARASOC cruise is a malacologist. On that account, it is likely that their networks of collaborators include more malacologists and carcinologists than specialists of other taxa. The time spent between sampling and species descriptions - the shelf life of specimens^44^- is thus highly contingent, and results in a bias in taxonomic coverage. For example, survey logbooks from MUSORSTOM 9 stated that large amounts of Echinoids and Asteroids were sampled^26^. Moreover, the field photos and the logbooks both confirm the presence of Cnidaria and Porifera in the sampled stations, but in very low abundances. These taxa are lacking in our dataset because none have been identified by a taxonomist yet. They include habitat-forming species which often dominate the biomass and shape the habitats^45^. As such, they are used as indicator taxa for Vulnerable Marine Habitats (VME) in the deep sea^46^. However, no coral gardens, sponge grounds, or crinoid fields, which have been described on some cobalt-rich seamounts in the Central Pacific^9,47^, were observed in the three surveys in French Polynesia. The impact of this taxonomic bias in our analysis is probably largely counterbalanced by the amount of data gathered for the Mollusca and the Crustacea.

Integrating our biological data with the distinct history and environmental conditions of the surveyed area^15,48^, reveals a pronounced geographic structure within the French Polynesian EEZ. Indeed, the three areas only shared very few species; most of the species were found only in one of the three cruises (Fig. 3c). Recent inventories in Polynesian shallow waters provide similar results. For example, the mapping of sponge species from 0 to 60 m depth over the different archipelagos showed that 46% of the species are limited to French Polynesia, and that 30% of the species are restricted to a single island^49^. Depth and substrate type are the other important factors known to structure deep-sea communities at different spatial scales^50–53^. Despite the intrinsic limitations of the data derived from naturalistic explorations, our analyses show that geography, depth, topography and the substrate type also structure the deep-sea communities of French Polynesia; both at the scale of the EEZ (Fig. 4a,b), and at the scale of the sub-region explored by the TARASOC cruise (see Fig. 2b).

Some recent studies, based on submarine imaging techniques, show that benthic communities on seamounts differ in response to the presence (or absence) of polymetallic crust^8^, or in response to different levels of hydrothermal activity^54^. Furthermore, the video-survey of the megafauna on cobalt-rich seamounts in the central Pacific, reveal the patchy, and heterogeneous distribution of the organisms, even at the scale of a single pinnacle^9^. In these studies, dense assemblages, dominated in biomass by suspension-feeding organisms like corals, sponges, or pedunculated crinoids, cover the surveyed environments. The analysis of the spatial distribution of such abundant and large organisms is possible using imagery data. But such techniques are not well-suited for surveying environments inhabited by small organisms living in low densities. In the largest known oceanic desert, the alternative sampling method, employing physical sampling of biological specimens and precise taxonomic identifications, reveals that polymetallic crusts, are inhabited by distinct macrobenthic communities. Submarine imaging techniques would certainly not have allowed us to obtain this result.

The analyses of the molluscan dataset allowed us to reveal the differences in size spectrum, between the communities living on seamounts or polymetallic crusts, and between the communities living on island slopes. We should stress that, had we considered only the available taxonomic literature, our analysis would not have revealed the size spectra. Indeed, for the Molluscs only 11 out of 48 articles considered for dataset #2, included description of micromolluscs. This bias in the published literature is due to the time needed for the identification of smaller molluscs^22,55,56^. Among the molluscs sampled during these three cruises, the smaller specimens are still mostly identified only at the family level.

In our dataset, the smaller size of molluscs on seamounts, in comparison to island slopes, could reflect the impoverishment in food input on isolated seamounts. Contrary to isolated seamounts, island slopes are supplied with terrestrial inputs. Abundance and biomasses of animals decrease with depth, but this decrease is more important for large animals than for small ones^57^. Deep-sea environments with unusually high food inputs (like hydrothermal vents or, animal or plant falls), have high abundances of large animals^58^. The limited data available tend to show that crusts are preferentially formed where the sedimentation rate is low^59^. In this area, the presence of crust might well be an indication of a low food input. The results we obtained by considering different size-spectra thus suggest that the communities occurring across the different habitats, differ not only in species diversity, but also in functional diversity.

## Conclusions

Deep-sea environments remain as one of the least known ecosystems. The data are scarce and it remains difficult to study diversity patterns, either in terms of species richness, or functional diversity. Species diversity and rarity can be studied through the presence of taxa. Applying Gaston’s rule on our occurrence data, we would consider that 40% of the present species are rare. Considering that the sampling is not saturated at all, this high percentage should however be considered cautiously. While part of this high level of rarity results from insufficient sampling, these results raise the question of the functional role of rarity in these environments, in quite remote area. The role of rare species in the functional structure of biological communities is still poorly understood, but rare species may endorse original functions and are more prone to extinction^60,61^. The loss of rare species should thus greatly impact ecosystem functioning. In this context, Mouillot *et al*. (2013) stated that, protecting species that are locally rare is as important as protecting species with a reduced occupancy area^62^. In French Polynesia, which is located right in the middle of the South Pacific region and is often considered as the largest oceanic desert, we show that the seamounts are not dominated by habitat-forming species (coral gardens, sponge grounds, etc), which are found elsewhere in the Pacific. These seamounts are however inhabited by unique communities. The analysis of the size range from Molluscan shells also shows a trend of smaller size shells occurring on seamounts and encrusted substrata. The differences in body sizes among these environments probably reflects the low food input associated with a low rate of sedimentation. These remote ecosystems are threatened by anthropogenic pressures such as mining^3^, but also climate change^63^. It should be emphasized that polymetallic crust exploitation is a tangible business option for French Polynesia^5^. Mining activities are generally associated with the release of residues, thus leading to an increased rate of sedimentation, which could affect biological communities that are adapted to low rates of sedimentation. Hence, considering the area as a desert with a high proportion of rare and small species, and differences in communities’ composition depending, notably, on the presence of crust, how should our results be considered for management and conservation purposes? We do not propose rigorous answers, but it is clear that the deep-waters of French Polynesia host unique benthic communities, and endemic species, and as such should be considered as sensitive environments^12^. Moreover, as these communities harbour many small sized organisms, it makes them hard to spot with submarine imaging techniques. Consequently, surveys based only on submarine imaging surveys (photos or videos) would miss the small fraction of the biodiversity in these type of environments, and therefore should not be considered as the only data acquisition option^64^. In contrast, the accurate identification of the entire material collected by the naturalist explorations of the TDSB program, will surely provide additional insights on the determinants of deep-sea benthic communities^65^. Moreover, it is fundamental to pursue the exploration of these immensely large areas in order to prevent species disappearing before being discovered^2,66^.

## Supplementary information

Supplementary Dataset 1

## Data Availability

The species datasets generated and analysed during the current study are available in the Supplementary Material.
